# Intravenous anesthetics have differential effects on human potassium channels: Interaction between anesthetics and potassium channels

**DOI:** 10.3724/abbs.2024151

**Published:** 2024-09-13

**Authors:** Ying Tao, Kejie Yao, Jing Wu, Tian Xu, Junhui Lin, Yi Qin, Diansan Su, Shiqing Cai, Weifeng Yu, Xuemei Chen

**Affiliations:** 1 Department of Anesthesiology Renji Hospital Shanghai Jiao Tong University School of Medicine Shanghai 200127 China; 2 Key Laboratory of Anesthesiology (Shanghai Jiao Tong University) Ministry of Education Shanghai 200127 China; 3 Institute of Neuroscience State Key Laboratory of Neuroscience CAS Center for Excellence in Brain Science and Intelligence Technology Chinese Academy of Sciences Shanghai 200031 China; University of Chinese Academy of Sciences Beijing 101408 China; 4 Department of Equipment and Materials Biomedical R&D Project Team Zhongshan Hospital Fudan University Shanghai 200031 China

**Keywords:** general anesthetics, hypotension, respiratory depression, potassium channels, molecular docking

## Abstract

General anesthetics are widely used in the clinic and greatly promote the development of surgery. However, the incidence of cardiovascular and respiratory complications caused by general anesthetics is still high, and the underlying mechanisms remain incompletely understood. Potassium channels are widely expressed in the heart and blood vessels and participate in regulating blood pressure, heart rate, and other physiological parameters. Whether they are directly affected by intravenous general anesthetics is unclear. Here, we independently express four classes of potassium channels, TASK-1, TASK-3, Kv1.5, Kv2.1, Kir2.1, SK1 and SK3, in *Xenopus* oocytes. The effects of propofol, pentobarbital and ketamine on these channels are evaluated by their current change. We find that propofol and ketamine potentiate TASK-3 and SK3 current respectively, while pentobarbital and ketamine inhibit SK1 current. To identify the key residues in TASK-3, SK1 and SK3 that interact with intravenous anesthetics, we predict homology models of the three channels and perform molecular docking simulations. The results show that propofol forms a hydrogen bond with Q126 of TASK-3, ketamine forms a hydrogen bond with S290 of SK1 and S467 of SK3, while pentobarbital forms hydrogen bonds with S330 and T358 of SK1. As these potassium channels are closely related to respiratory system regulation, cardiac rhythm and vasodilation, our study provides a new perspective for further study on the mechanism of general anesthetics-induced respiratory and circulatory side effects.

## Introduction

Over 170 years have passed since general anesthetics were first used to induce anesthesia during surgery. The extensive use of intravenous general anesthetics has greatly accelerated the development of surgery. However, a number of adverse effects, particularly those related to the cardiovascular and respiratory systems, limit the use of general anesthetics. Numerous studies have reported that patients treated with propofol can experience severe bradycardia arrhythmias [ 1, 2], respiratory inhibition [ 3, 4], and hypotension induced by vascular resistance reduction [ 5‒ 7]. Studies have shown that ketamine, a commonly used anesthetic in pediatric surgery, can directly act on vascular endothelial cells to promote vasodilation, which results in hypotension in shock patients [8]. Additionally, pentobarbital decreases heart rate [9]. Maintaining the stability of the cardiovascular and respiratory systems during surgery has a great impact on the smooth operation and postoperative prognosis of patients. However, the exact mechanism of the cardiovascular and respiratory system effects of these general anesthetics remains unclear.

Potassium channels are widely expressed in the heart and vascular cells, where they help regulate cardiac conduction and myogenic tone [10]. Previous studies have reported that inhalational anesthetics can influence the currents of several potassium channels in cultured primary cells or in recombinant systems. Potassium channels may also be targets of general anesthetics and can be highly relevant to cardiovascular and respiratory side effects.

Previous studies have shown that potassium channels associated with general anesthetics can be categorized into four groups: voltage-gated (Kv) channels, background/leak or tandem 2-pore (K2P) families, inwardly rectifying (Kir) channels, and Ca2 ^+^-activated (KCa) channels [11]. Here, we selected several potassium channels, which are highly expressed in heart or vascular cells, including the Kv1.5, Kv2.1, TASK-1, TASK-3, Kir2.1, SK1 and SK3 channels. These channels have been reported to regulate cardiac rhythm or blood pressure. We expressed them in *Xenopus* oocytes to study whether propofol, ketamine and pentobarbital could bind to these channels and cause current changes.

Our results showed that the expressions of TASK-1, Kv1.5, Kv2.1 and Kir2.1 in oocytes could not be affected by the three intravenous general anesthetics, while the anesthetics had varying degrees of effect on the currents of TASK-3, SK1 and SK3. As TASK-1, SK1 and SK3 play different roles in cardiac conduction and myogenic tone, they may partially explain the diverse side effects of these anesthetics. In addition, we predicted the protein structures of TASK-3, SK1 and SK3 and carried out molecular docking with propofol, pentobarbital and ketamine to predict the possible binding sites of small molecules and channels.

This is the first time to report intravenous anesthetics directly interacting with TASK-3, SK1 and SK3. Our study may contribute to future researches which aim to address the respiratory and cardiovascular side effects of general anesthetics.

## Materials and Methods

### Construct design

For electrophysiological experiments, the full human genes *KCNA5* (NP_002225), *KCNB1* (NP_004966), *KCNJ2* (NP_000882), *KCNK3* (NP_002237), *KCNK9* (NP_001269463), *KCNN1* (NP_002239), and *KCNN3* (NP_002240), which encode the Kv1.5, Kv2.1, Kir2.1, TASK-1, TASK-3, SK1 and SK3 channels, respectively, were cloned by PCR using the primer: *Kv1*. *5*, forward 5′-ATGGAGATCGCCCTGGTG-3′, reverse 5′-TCACAAATCTGTTTCCCGGCT-3′; *Kv2*. *1*, forward 5′-ATGCCGGCGGGCATGACGAA-3′, reverse 5′-TCAGATGCTCTGATCTCGTGTG-3′; *Kir2*. *1*, forward 5′-ATGGGCAGTGTGCGAACC-3′, reverse 5′-TCATATCTCCGACTCTCGCCG-3′; *TASK-1*, forward 5′-ATGAAGCGGCAGAACGTG-3′, reverse 5′-AGTCACACGGAGCTCCT-3′; *TASK-3*, forward 5′-ATGAAGAGGCAGAACGTGCG-3′, reverse 5′-CTAAACGGACTTCCGGCGTT-3′; *SK1*, forward 5′-ATGAACAGCCACAGCTACAA-3′, reverse 5′-AAGATTTAGGGGTCTGGTGGC-3′; *SK3*, forward 5′-ATGGACACTTCTGGGCAC-3′, reverse 5′-TTAGCAACTGCTTGAACTTGTGTAC-3′, and then cloned into pcDNA3-based vectors. cRNA was transcribed *in vitro* with mMESSAGE mMACHINE™ T7 ULTRA (Thermo Fisher Scientific, Waltham, USA).

### Drugs and chemicals

Propofol (MCE, Monmouth Junction, USA) was dissolved in dimethyl sulfoxide (DMSO; Sigma-Aldrich, St Louis, USA) to prepare a stock solution (100 mM). The final dose of DMSO was ≤0.1%. Pentobarbital (Sigma-Aldrich) was dissolved in recording solution. Ketamine was diluted from a ketamine hydrochloride injection (50 mg/mL) obtained from the Center for Excellence in Brain Science and Intelligence Technology (Shanghai, China). The oocytes were incubated in drugs for 5 min to test the effect.

### Oocyte isolation and cRNA injection

All procedures involving animal experiments were complied with the ARRIVE guidelines and were carried out in accordance with the UK Animals (Scientific Procedures) Act, 1986 and associated guidelines.

Oocytes were obtained from mature female *Xenopus laevis* and incubated in OR2 solution (85 mM NaCl, 5 mM HEPES, and 1 mM MgCl _2_, pH 7.6 with KOH). After treatment with collagenase II (Sigma-Aldrich), the oocytes were each injected with 10‒25 ng of cRNAs and incubated in Barth solution (88 mM NaCl, 10 mM HEPES, 1 mM KCl, 2.4 mM NaHCO _3_, 0.33 mM Ca(NO _3_) _2_, 0.41 mM CaCl _2_, and 0.82 mM MgSO _4_, pH 7.6 with NaOH). All the oocytes were stored in an incubator at 16°C.

### Two-electrode voltage clamp recordings

The currents of the Kir2.1, Kv2.1, Kv1.5, TASK-1 and TASK-3 channels were measured by two-electrode voltage-clamp (TEVC) recording 1 day after injection in ND96 solution (96 mM NaCl, 5 mM HEPES, 2 mM KCl, 1.8 mM CaCl _2_, and 1 mM MgCl _2_, pH 7.6 with NaOH). Oocytes were held at a potential of ‒60 mV, and the channel currents were elicited by 2 s pulses in 10 mV or 20 mV increments. The currents of the SK1 and SK3 channels were recorded 3 days after injection in high-K ^+^ solution (98 mM KCl, 0.3 mM CaCl _2_, 1 mM MgCl _2_, and 5 mM HEPES, pH 7.6 with NaOH). The voltage was held at 0 mV and increased from ‒140 mV or ‒120 mV to 0 mV in 20 mV increments. Electrophysiology data were collected and analyzed using pClamp10 (Molecular Devices, Sunnyvale, USA) and Clampfit.

### Autodock

We obtained the structures of TASK-3, SK1 and SK3 using the SWISS-MODEL homology-modelling server ( https://swissmodel.expasy.org/). We used TASK-1 as a homology model for constructing TASK-3 structure and SK4 for SK1 and SK3. Molecular docking was performed by using CB-DOCK2 online server ( https://cadd.labshare.cn/cb-dock2/php/index.php) auto blind docking. Non-covalent protein-ligand interactions were analyzed with PLIP ( https://plip-tool.biotec.tu-dresden.de/plip-web/plip/index) and LigPlot+.

### Data analysis

Data processing and statistical analysis of TEVC were conducted using GraphPad software (GraphPad, San Diego, USA), and data are reported as the mean±SD. Dose-response curves were fitted to the variable slope of the Hill equation to calculate the IC _50_ or EC _50_. The maximum or minimum responses were constrained to 1 for the curve fitting. The Hill equation was as follows: response=1+(Top–1)/(1+10^((LogEC _50_–LogConcentration)×HillSlope)) or response=Bottom+(1–Bottom)/(1+10^((LogIC _50_–LogConcentration)× HillSlope)). All recordings were repeated with more than 3 batches of oocytes. As the currents of some channels could vary among oocytes, currents were normalized to the value obtained at the voltage with the largest amplitude under control conditions and are shown as the normalized current. For all recordings, Student’s *t* test was used, and *P*<0.05 was considered statistically significant.

## Results

### Propofol activates TASK-3 current in oocytes

Two-pore-domain K ^+^ (K2P) channels conduct cardiac outward K ^+^ currents, stabilize the resting membrane potential and facilitate action potential repolarization [ 12, 13]. TASK-1 and TASK-3 are highly expressed in the heart of mammals [ 14, 15]. Previous studies have shown that some inhaled anesthetics can activate TASK-1 and TASK-3 [ 16, 17], but etomidate inhibits them [18]. Here, we studied the effect of intravenous anesthetics, including propofol, pentobarbital and ketamine, on TASK-1 and TASK-3.

**Figure 1:**
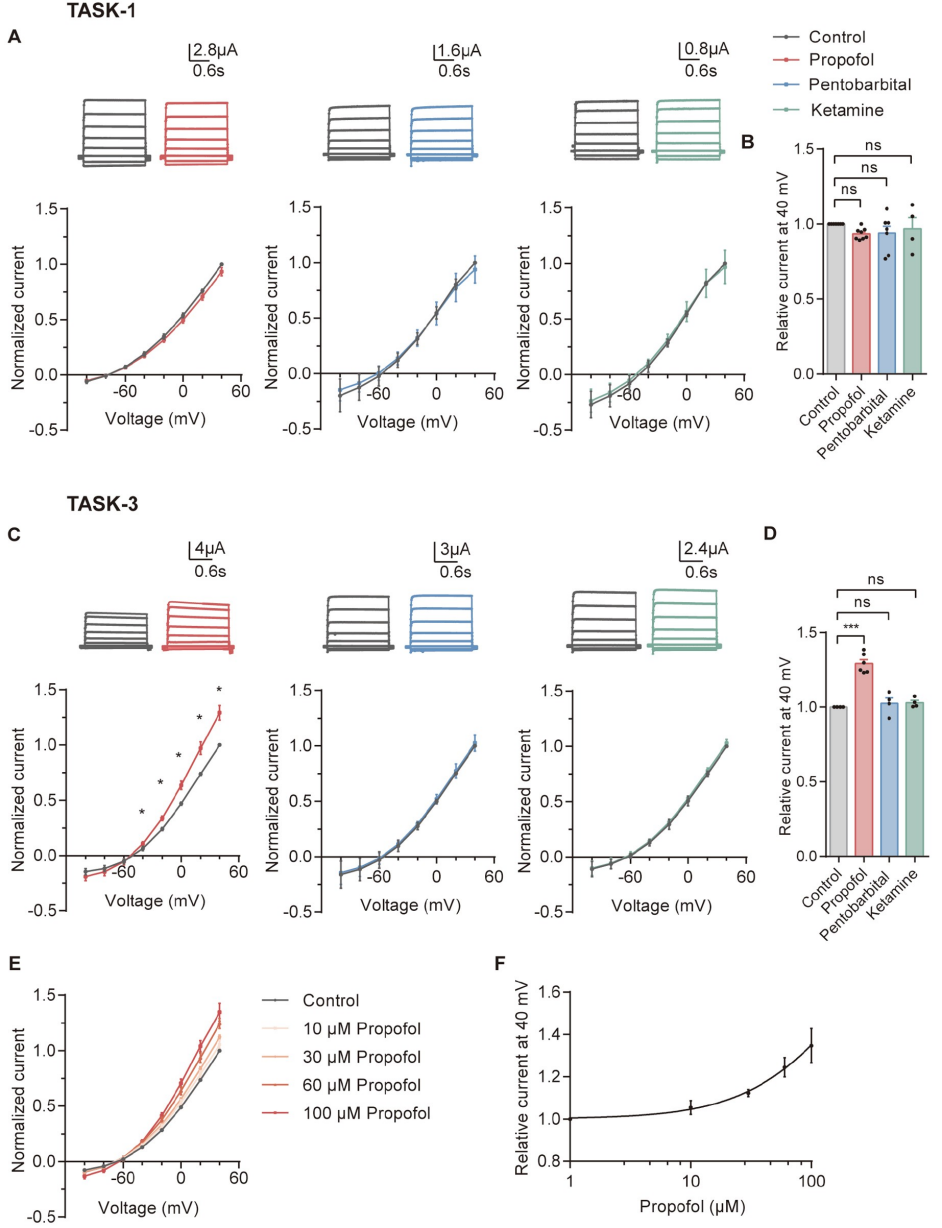
Figure 1 Dose-dependent activation of TASK-3 by propofol Representative recording traces of TASK-1 (A, top) and TASK-3 (C, top) in the control condition and in the presence of 100 μM propofol, 100 μM pentobarbital and 90 μM ketamine. The holding potential was ‒60 mV. The voltage was increased from ‒100 to +40 mV in 20-mV increments. Normalized I‒V relationships of TASK-1 (A, bottom) and TASK-3 (C, bottom) for step currents measured at the end of the test pulses in the absence (control) and in the presence of 100 μM propofol (nTASK-1=8; nTASK-3=6), 100 μM pentobarbital (nTASK-1=7; nTASK-3=4) and 90 μM ketamine (nTASK-1=4; nTASK-3=4). Currents were normalized to the amplitude at +40 mV under control conditions. Summary of the normalized data at +40 mV for TASK-1 (B) and TASK-3 (D) measured at the end of the test pulses in the absence (control) and in the presence of 100 μM propofol, 100 μM pentobarbital and 90 μM ketamine. I-V relationships of TASK-3 at 10, 30, 60, and 100 μM propofol (E, n=6) and the dose‒response curve (F) at ‒140 mV. *P<0.05; ***P<0.001; ns, not significant.

To test the effect on TASK-1, the current traces were recorded in oocytes using TEVC. The recording was obtained in ND96 solution (pH 7.6), and the holding potential was ‒60 mV. The voltage was increased from ‒100 to +40 mV in 20-mV increments. Figure 1A shows representative current-voltage trace of TASK-1 recorded before and after the application of 100 μM propofol, 100 μM pentobarbital or 90 μM ketamine for 5 min. The traces of blank oocytes injected with water showed that currents were induced by TASK-1 and TASK-3 (data not shown). The current-voltage curves and normalized currents showed no significant differences in the presence of these three general anesthetics ( Figure 1A,B), which indicated that propofol, ketamine and pentobarbital had no direct interaction with TASK-1.

For TASK-3, the recording protocol was the same as that for TASK-1. Ketamine and pentobarbital had no significant effect on TASK-3 expression in oocytes. In contrast, 100 μM propofol activated the TASK-3 current ( Figure 1C). The current significantly increased from ‒20 mV to +40 mV after incubation with propofol for 5 min, as shown by the normalized current. The current at +40 mV increased by approximately 25% ( Figure 1D). We also tested the currents under 10, 30 and 60 μM propofol and found that it had a dose-dependent effect ( Figure 1E,F). Considering that higher concentrations of DMSO could influence oocytes, we did not test currents under higher concentrations of propofol.

### Kv channels are insensitive to propofol, ketamine and pentobarbital

The interactions of Kv1.5 and Kv2.1 with inhalational anesthetics have been widely studied. Previous studies have shown that sevoflurane influences the current of Kv1.5 [ 19, 20]. Halothane can inhibit Kv2.1 [21]. Both the mRNA and protein expressions of the Kv1.5 and Kv2.1 channels have been detected in various vascular tissues [ 22, 23]. Therefore, here, we tested whether general anesthetics could directly influence Kv1.5 and Kv2.1 in heterologous expression system.

**Figure 2:**
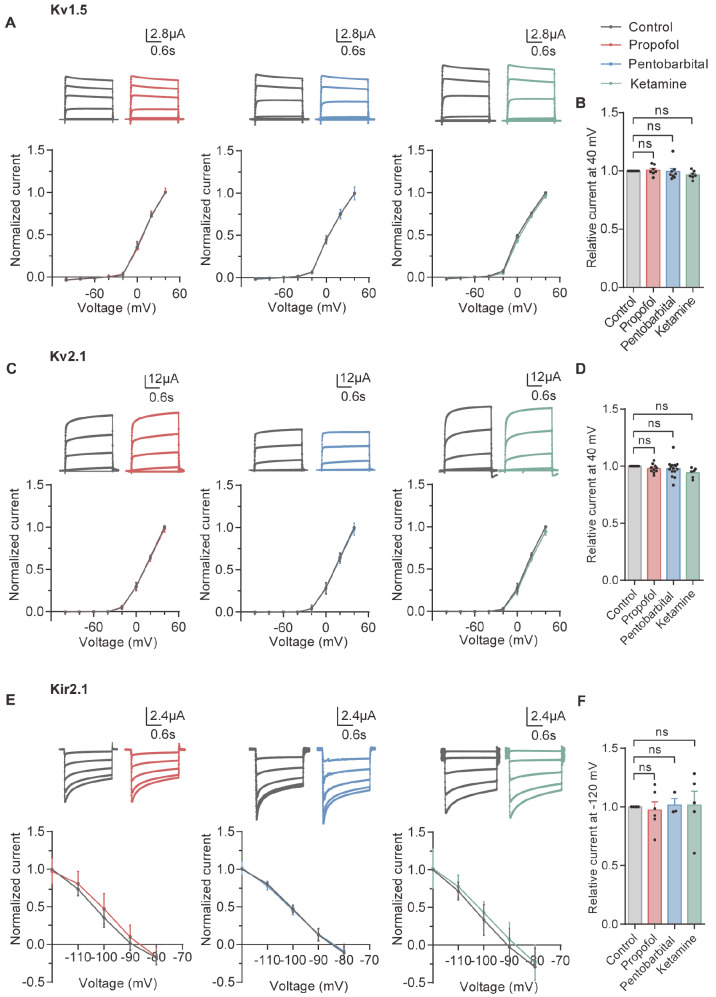
Figure 2 Propofol, ketamine and pentobarbital have no effect on Kv1.5, Kv2.1, or Kir2.1 Representative recording traces of Kv1.5 (A, top), Kv2.1 (C, top) and Kir2.1 (E, top) in the control condition and in the presence of 100 μM propofol, 100 μM pentobarbital and 90 μM ketamine. Normalized I‒V relationships of Kv1.5 (A, bottom), Kv2.1 (C, bottom) and Kir2.1 (E, bottom) for step currents measured at the end of the test pulses in the absence (control) and in the presence of 100 μM propofol (nKv1.5=7; nKv2.1=10; nKir2.1=6), 100 μM pentobarbital (nKv1.5=8; nKv2.1=15; nKir2.1=3) and 90 μM ketamine (nKv1.5=7; nKv2.1=6; nKir2.1=5). Currents were normalized to the amplitude of the current recorded at +40 mV for Kv1.5 and Kv2.1 and at ‒120 mV for Kir2.1 under the control condition. Summary of the normalized data at +40 mV for Kv1.5 (B) and Kv2.1 (D) and ‒120 mV for Kir2.1 (F) measured at the end of the test pulses in the absence (control) and presence of 100 μM propofol, 100 μM pentobarbital and 90 μM ketamine. ns, not significant.

The results showed that propofol, ketamine and pentobarbital had no effect on the currents of Kv1.5 or Kv2.1 from ‒100 mV to +40 mV, as shown by the current‒voltage curves and normalized I‒V relationships ( Figure 2A,C). The mean current rate showed that ketamine slightly inhibited Kv1.5 at +40 mV, but the difference was not significant ( Figure 2B,D). This indicated that drugs may not cause side effects by directly affecting Kv1.5 and Kv2.1 channels.

### Propofol, ketamine and pentobarbital have no significant effects on Kir2.1

Kir2.1 channels exhibit robust expression in ventricles [24] and smooth muscle cells [ 25‒ 28]. These channels contribute to the major inward K ^+^ current in ventricular and atrial myocardial cells [ 29, 30]. Previous research has shown that the local anesthetics bupivacaine and lidocaine can inhibit Kir2.x channels [31]. We tested the influence of intravenous general anesthetics on Kir2.1.

We recorded the current using the same solution as described above, and the voltage was increased from ‒80 to ‒120 mV in increments of ‒10 mV. Ketamine, propofol and pentobarbital did not directly influence Kir2.1 ( Figure 2E,F).

### Ketamine and pentobarbital inhibit SK1, while ketamine activates SK3

Small-conductance calcium-activated potassium (SK) channels are expressed in atrial, ventricular and conducting system cardiomyocytes and are associated with atrial fibrillation and arrhythmogenesis [ 32, 33]. However, the interaction of SK channels with anesthetics has rarely been studied. Here, we studied the effects of intravenous anesthetics on SK1 and SK3 channels.

**Figure 3:**
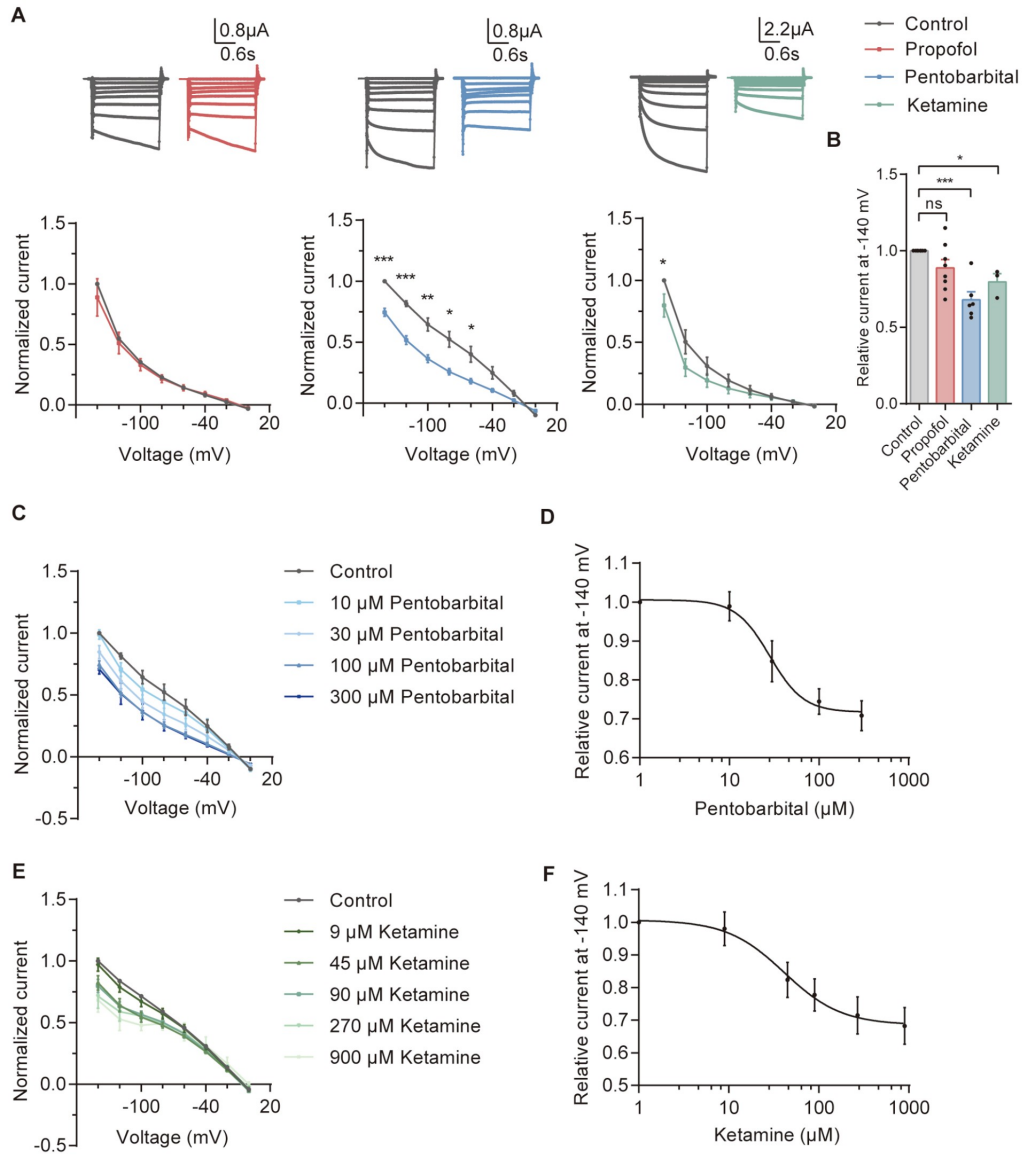
Figure 3 Ketamine and pentobarbital inhibit the SK1 channel Representative recording traces of SK1 (A, top) in the control condition and in the presence of 100 μM propofol, 100 μM pentobarbital and 90 μM ketamine. Normalized I‒V relationships of SK1 (A, bottom) for step currents measured at the end of the test pulses in the absence (control) and in the presence of 100 μM propofol (n=8), 100 μM pentobarbital (n=6) and 90 μM ketamine (n=7). Currents were normalized to the amplitude of the current recorded at ‒140 mV for SK1 under the control condition. Summary of the normalized data at ‒140 mV for SK1 (B) measured at the end of the test pulses in the absence (control) and presence of 100 μM propofol, 100 μM pentobarbital and 90 μM ketamine. Normalized I‒V relationships of SK1 at 10, 30, 100, and 300 μM pentobarbital (C, n=3) and 9, 45, 90, 270, and 900 μM ketamine (E, n=4) and dose‒response curves of pentobarbital (D, IC50=36.87±22.68 μM) and ketamine (F, IC50=51.97±23.37 μM) at ‒140 mV. *P<0.05; **P<0.01; ***P<0.001; ns, not significant.

The current was recorded in a high K ^+^ solution. For SK1, the voltage was held at 0 mV and increased from ‒140 mV to 0 mV in 20 mV increments. However, 100 μM propofol had no effect on SK1 channels. Interestingly, 90 μM ketamine and 100 μM pentobarbital inhibited the SK1 current ( Figure 3A). The mean current rate at ‒140 mV showed that ketamine and pentobarbital inhibited the current of SK1 by approximately 30% ( Figure 3B). The dose‒response curves of pentobarbital ( Figure 3C) and ketamine ( Figure 3E) are shown. The IC _50_ values of pentobarbital and ketamine were 36.87±22.68 μM ( Figure 3D) and 51.97±23.37 μM ( Figure 3F), respectively. Ketamine and pentobarbital had little effect on SK1 at a concentration of 10 μM.

**Figure 4:**
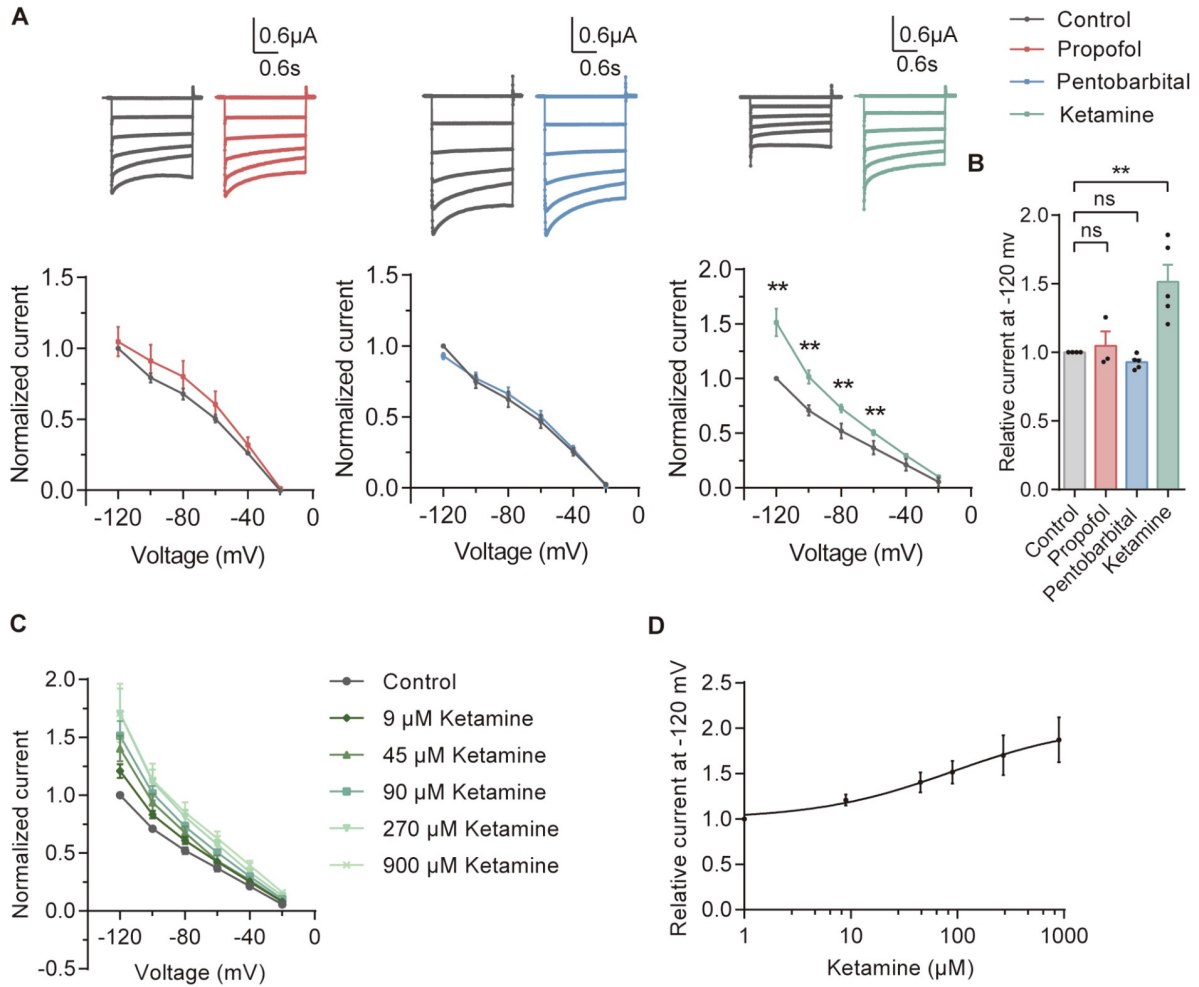
Figure 4 Dose-dependent activation of the SK3 channel by ketamine Representative recording traces of SK3 (A, top) in the control condition and in the presence of 100 μM propofol, 100 μM pentobarbital and 90 μM ketamine. Normalized I‒V relationships of SK3 (A, bottom) for step currents measured at the end of the test pulses in the absence (control) and in the presence of 100 μM propofol (n=3), 100 μM pentobarbital (n=5) and 90 μM ketamine (n=5). Currents were normalized to the amplitude of the current recorded at ‒120 mV under the control condition. Summary of the normalized data at ‒120 mV for SK3 (B) measured at the end of the test pulses in the absence (control) and in the presence of 100 μM propofol, 100 μM pentobarbital and 90 μM ketamine. Normalized I‒V relationships of SK3 at 9, 45, 90, 270, and 900 μM ketamine (C, n=5) and the dose‒response curve (D, EC50=56.87±12.60 μM) at ‒120 mV. **P<0.01; ns, not significant.

We also found that 90 μM ketamine potentiated the SK3 current at all voltages tested ( Figure 4A) and increased the current at ‒120 mV by approximately 40% ( Figure 4B). We also recorded SK3 currents after treatment with 9, 45, 90, 270 and 900 μM ketamine ( Figure 4C). The dose‒response curve showed that the IC _50_ of ketamine for the SK3 channel was 56.87±12.60 μM ( Figure 4D).

### Prediction of the binding sites of general anesthetics with potassium channels by autodock

**Figure 5:**
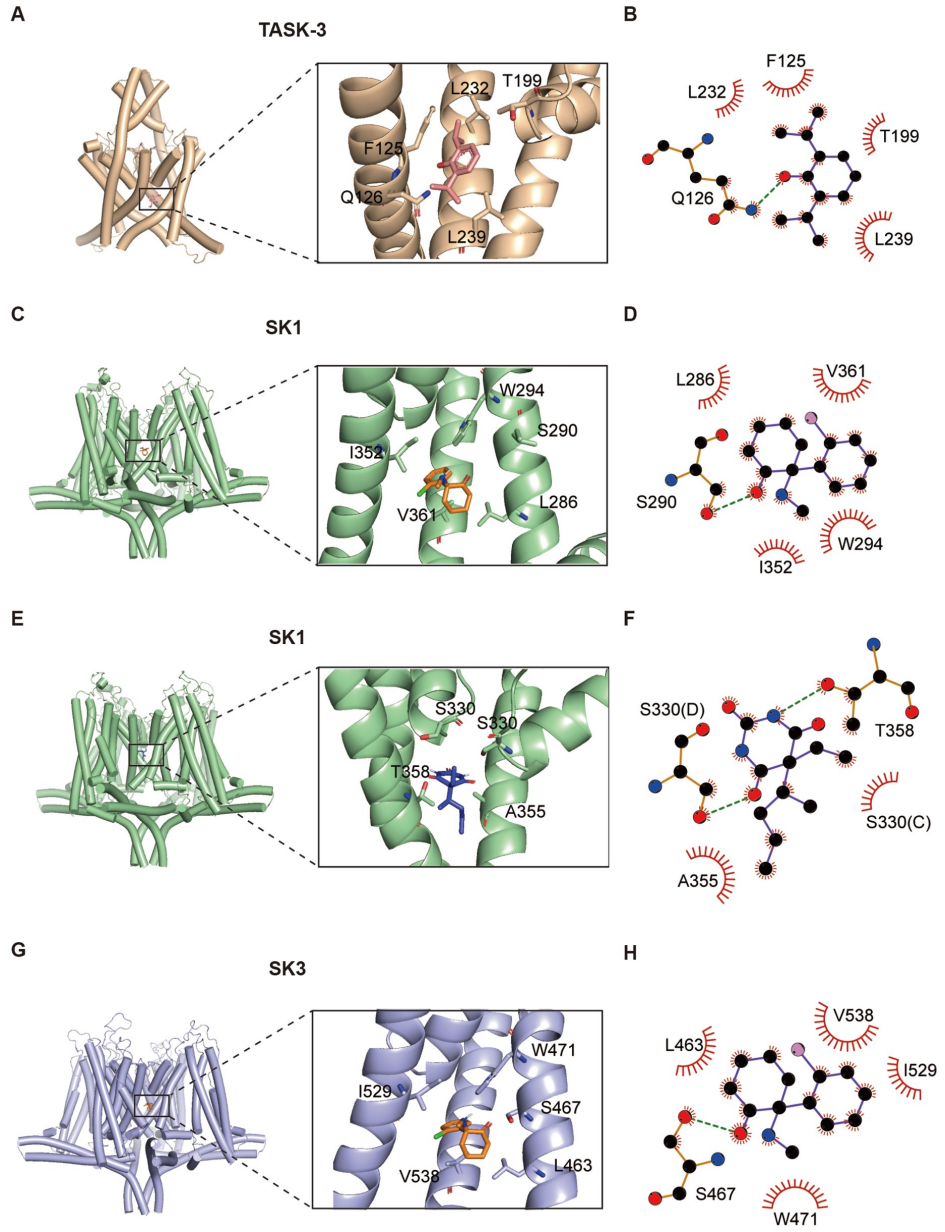
Figure 5 The predicted protein structures of TASK-3, SK1, SK3 and molecular docking of ketamine and pentobarbital within these channels The predicted protein structures of TASK-3 (A, left), SK1 (C, left; E, left), and SK3 (G, left) were modeled using SWISS-MODEL. Molecular docking of propofol within the inner cavity of the TASK-3 channel (A, right), ketamine with the SK1 channel (C, right), pentobarbital with the SK1 channel (E, right) and ketamine with the SK3 channel (G, right). The LIGPLOT program was used to generate a schematic depiction of the main interactions of propofol with the TASK-3 channel (B), ketamine with the SK1 channel (D), pentobarbital with the SK1 channel (F) and ketamine with the SK3 channel (H). A distance between the donor and acceptor of less than 3.5 Å is considered a hydrogen bond, and a distance of 4.1 Å between two hydrophobic atoms is considered a hydrophobic interaction.

To study the potential binding sites of general anesthetics with those potassium channels, we performed molecular docking. The structures of TASK-3, SK1 and SK3 were obtained from the SWISS-MODEL homology-modeling server. Molecular docking was performed by using the CB-DOCK2 online server ( Figure 5A‒D). The results showed that propofol might bind to the transmembrane domain of TASK-3. It has hydrophobic interactions with L232, F125, T199 and L239. The phenolic hydroxyl group on propofol forms a hydrogen bond with Q126 ( Figure 5E). The SK1 and SK3 channels have similar structures because of their high homology. The auto blind docking showed that ketamine might bind to the same cavity of SK1 and SK3 channels. In this cavity, ketamine interacts with tryptophan, isoleucine, valine, serine, and lysine.

The carbonyl group of ketamine on cyclohexane binds to S290 of SK1 and S467 of SK3 via a hydrogen bond ( Figure 5F,H). Pentobarbital looks like a pore blocker of SK1. The binding site is near the selectivity fitter. Pentobarbital has hydrogen bonds with S330 and T358 ( Figure 5G). Together, these data indicate that the three intravenous anesthetics bind to potassium channels.

## Discussion

Respiratory and circulatory depression caused by general anesthesia is a serious complication during surgery, but the exact mechanism remains unclear. Here, we identified several different types of potassium channels associated with the cardiovascular and respiratory systems in *Xenopus* oocytes and found that propofol can directly modulate the TASK-3 current instead of affecting Kv1.5, Kv2.1, Kir2.1, SK1, SK2 and TASK-1, and the other two intravenous general anesthetics, ketamine and pentobarbital, inhibited the SK1 current, while ketamine potentiated the SK3 current. In addition, the possible docking sites of these molecules with general anesthetics were predicted by using the CB-DOCK2 online server. This study identified potential targets for circulatory and respiratory depression caused by intravenous anesthetics, which may provide new perspectives for improving the safety of anesthesia.

In this work, we found that 100 μM propofol could potentiate the TASK-3 current by approximately 25%. Previous research has demonstrated that 100 μM propofol is unable to affect the TASK-3 current [18]. This inconsistency may arise due to the following factors: (1) We exposed oocytes to propofol for a more extended incubation period to ensure better integration, as the binding kinetics of propofol may be low or because propofol binds to the transmembrane and intracellular domains of the channels. (2) The sample size was smaller in previous studies, and the significance of differences may be missed. TASK-3 is highly expressed in the heart and blood vessels, where it conducts background currents in atrial cells and regulates cardiac rhythm [34]. Moreover, TASK-3-deficient mice exhibit cardiac hypertrophy and hypertension phenotypes [ 35, 36]. It has also been reported that TASK-3 might be a molecular target for antiarrhythmic drugs [37]. Taken together, propofol may slow the cardiac rhythm and induce hypotension through this channel. In addition, TASK-3 is also expressed in carotid body type 1 glomus chemo-sensing cells, which play an essential role in respiratory system regulation. TASK-3 antagonists stimulate respiration in rodents [38]. Thus, it is possible for propofol to inhibit respiration by activating the TASK-3 channel in the carotid body.

Our results also suggested that the SK1 current is inhibited by ketamine and pentobarbital. SK1 was reported to conduct background currents in atrial cells and regulate cardiac rhythm, and either increased or decreased SK1 expression causes clinical atrial and ventricular arrhythmogenesis [ 33, 37]. Thus, ketamine and pentobarbital might modulate cardiac rhythm by inhibiting SK1. However, in the clinic, pentobarbital slows the cardiac rhythm, while ketamine accelerates it. The reason is unknown, and it may be caused by the different affinities and kinetics of these two molecules, which needs to be further studied.

In contrast to SK1, ketamine activates SK3 channels in a dose-dependent manner. The activation of endothelial SK3 causes arteriolar dilation by disrupting the endothelium-derived hyperpolarizing factor vasodilator pathway [39]. Moreover, SK3 knockout mice are hypertensive, and increased blood pressure is also induced by impaired vasorelaxation [40]. Furthermore, *in vitro* experiments on canine basilar arteries showed that ketamine relaxes high-K ^+^-induced basilar artery contraction [41]. Our results showed that ketamine could activate SK3 in oocytes, which indicates that the SK3 channel may be the target of the hypotension induced by ketamine.

Although SK1 and SK3 are similar in structure, ketamine potentiates SK3 while inhibits SK1 and pentobarbital only inhibits SK1. The reason for this difference is complex, and we think that although the amino acids that interact with these two drugs on SK1 and SK3 are conserved, the discrepant domains may influence the pathway through which the molecules enter the binding pockets. In addition, posttranslational modifications may also affect the conformation of these proteins. To help address this problem, high-resolution structure analyses are needed in the future.

In addition, the IC _50_/EC _50_ of the three anesthetics to potassium channels are higher than 50 μM, suggesting that their affinities were relatively low. The plasma concentration of propofol, ketamine and pentobarbital during anesthesia is approximately 10 μM, indicating that anesthetic agents have little effect on these potassium channels at normal anesthetic concentrations. Given that side effects tend to occur at high doses, we think it is also reasonable that these anesthetics have a lower affinity for potassium channels.

For Kv2.1, our results showed that anesthetics could not influence the current of Kv2.1 expressed on oocytes, but previous studies have reported that propofol could inhibit the delayed rectifier potassium current which was induced by Kv2.1 in rat parietal cortical neurons [42]. This finding suggested that the delayed rectifier potassium current caused by propofol is not due to the direct binding of propofol with Kv2.1 channel.

In conclusion, our research is the first to report that commonly used intravenous anesthetics can specifically bind to certain potassium channels and affect their function. This study provides new insights into the potential mechanisms underlying cardiovascular system complications induced by general anesthetics, which may also provide ideas for therapeutic strategies for these serious clinical complications.
